# Endothelial Progenitor Cells Dysfunction and Senescence: Contribution to Oxidative Stress

**DOI:** 10.2174/157340308786349435

**Published:** 2008-11

**Authors:** Toshio Imanishi, Hiroto Tsujioka, Takashi Akasaka

**Affiliations:** Department of Cardiovascular Medicine, Wakayama Medical University, 811-1, Kimiidera, Wakayama City, Wakayama 641-8510, Japan

**Keywords:** Endothelial progenitor cell, oxidative stress, senescence, angiotensin II, telomerase, nitric oxide.

## Abstract

The identification of endothelial progenitor cells (EPCs) has led to a significant paradigm in the field of vascular biology and opened a door to the development of new therapeutic approaches. Based on the current evidence, it appears that EPCs may make both direct contribution to neovascularization and indirectly promote the angiogenic function of local endothelial cells *via *secretion of angiogenic factors. This concept of arterial wall repair mediated by bone marrow (BM)-derived EPCs provided an alternative to the local “response to injury hypothesis” for development of atherosclerotic inflammation. Increased oxidant stress has been proposed as a molecular mechanism for endothelial dysfunction, in part by reducing nitric oxide (NO) bioavailability. EPCs function may also be highly dependent on a well-controlled oxidant stress because EPCs NO bioavailability (which is highly sensitive to oxidant stress) is critical for their *in vivo *function. The critical question is whether oxidant damage directly leads to an impairment in EPCs function. It was revealed that activation of angiotensin II (Ang II) type 1 receptor stimulates nicotinamide-adenine dinucleotide phosphate (NADPH) oxidase in the vascular endothelium and leads to production of reactive oxygen species. We observed that Ang II accelerates both BM- and peripheral blood (PB)-derived EPCs senescence by a gp91phox-mediated increase of oxidative stress, resulting in EPCs dysfunction. Consistently, both Ang II receptor 1 blockers (ARBs) and angiotensin converting enzyme (ACE) inhibitors have been reported to increase the number of EPCs in patients with cardiovascular disease. In this review, we describe current understanding of the contributions of oxidative stress in cardiovascular disease, focusing on the potential mechanisms of EPCs senescence.

## INTRODUCTION

The identification of endothelial progenitor cells (EPCs) has led to a significant paradigm shift in the field of vascular biology. It has become evident that bone marrow (BM)-derived circulating EPCs can contribute to and amplify neovascularization. EPCs significantly contribute to adult vessel formation by physically incorporating and promoting vessel growth by paracrine mechanisms [1, 2]. It is believed that the majority of EPCs originate in the BM. The odyssey from BM to vascular endothelium can be divided into three stages. First, EPCs are mobilized or released from BM into systemic circulation in response to specific stimuli (mobilization). A number of cytokines and growth factors appear to promote this step. Subsequently, the cells appear to home to preferentially to sites of tissue injury (homing). Finally, some EPCs are incorporated into new blood vessels formed by the extension of existing vessels (angiogenesis) or possibly formed *in situ* (vasculogenesis). The concept of arterial wall repair mediated by BM-derived EPCs provided an alternative to the local “response to injury hypothesis” for the development of atherosclerotic inflammation. Based on the current evidence, it appears that EPCs may directly contribute to neovascularization and indirectly promote the angiogenic function of local endothelial cells *via* secretion of angiogenic factors. According to this new theory, the arterial wall can deal fairly well with multiple circulating and local noxious stimuli, as long as the BM-derived repair capacity, which induces competent EPCs and probably progenitors of other lineages, remains intact. Circulating EPCs are also indicators of overall cardiovascular health. Vasa *et al*. [3] initially showed that levels of circulating EPCs (CD34/kinase insert domain receptor (KDR)-positive) are higher in healthy volunteers than in patients with coronary artery disease. Hill *et al*. [4] analyzed “colony-forming units” of EPCs and found that this measurement negatively correlated with Framingham risk factor score. They also found that a reduction in EPCs colonies was a good predictor of impairment in flow-mediated brachial-artery reactivity. In a related study, it was reported that EPCs isolated from patients with type 2 diabetes mellitus display impaired proliferation and reduced incorporation into tube-like structures on Matrigel [5]. Conversely, statin (HMG-CoA reductase inhibitor) therapy increases circulating EPCs levels in patients with coronary artery disease [6]. Futhermore, an age-dependent decrease in EPCs mobilization has been reported [7]. Several of coronary risk factors are clustered in patients with cardiovascular disease. All of these risk factors are also associated with elevated markers of reactive oxygen species (ROS) [8].

The tissue microenvironment after ischemia is characterized by excessive production of ROS and oxidized metabolites. Prior studies have demonstrated that oxidative stress directly contributes to endothelial dysfunction and vascular disease [9-12]. These observations suggest that EPCs, in contrast to mature endothelial cells, are uniquely equipped with antioxidant defence systems to resist ROS-driven cytotoxicity if they are active participants in various repairs in ischemic tissues. Recently, two *in vitro* studies demonstrated that EPCs express higher levels of manganese superoxide dismutase (MnSOD) and glutathione peroxidase-1 (GPx-1) [13, 14]. It has also been shown that the collective inhibition of catalase, MnSOD, and GPx-1 increases ROS levels in EPCs and that this inhibition impairs EPCs survival and migration [15]. In fact, some studies have suggested that EPCs may be resistant to oxidative stress [15, 16]. Dernbach *et al*. [16] reported that EPCs are resistant to oxidative stress and uniquely equipped to repair damaged vessels in ischemic tissues. Some controversy does exist about the effects of oxidative stress on EPCs. Ingram *et al*. [17] reported that EPCs exhibited increased apoptosis and diminished tube-forming ability *in vitro* and *in vivo* in response to oxidative stress, which was directly linked to activation of a redox-dependent stress-induced kinase pathway. The current review describes the characterstics and properties of EPCs, focusing on the effects of oxidative stress on EPCs senescence.

## EPCs DEFINITION AND CHARACTERIZATION

The ability of the BM to give rise to endothelial cells was first reported by Asahara *et al* [1]. This study was based on the finding that EPCs circulating in peripheral blood (PB) express the hematopoietic marker CD34. The EPCs were defined as cells positive for both hematopoietic stem cells and endothelial cell markers, such as CD34 and vascular endothelial growth factor (VEGF) receptor-2, respectively. The latter VEGF receptor-2 is often referred to as KDR.

The putative CD34^+^ EPCs are able to proliferate and differentiate to mature endothelial cells with expression of different endothelial markers such as KDR [2, 18], platelet-endothelial cell adhesion molecule (CD31) [2, 15], von Willebrand factor [2, 18, 20], VE-cadherin [2, 18], caveolin-1 [19, 21], and endothelial nitric oxide (NO) synthase (eNOS) [19, 21]. While *in vitro*, EPCs can form vascular-like structures [18, 21], and *in vivo*, incorporate into neovessels at sites of tissue ischemia [18, 20, 22]. Of note, CD34 antigen density is the highest on early progenitors and decreases progressively as the cells mature [23]; however, CD34 is expressed not only on EPCs but on mature endothelial cells, albeit at a lower density [24]. As noted, it remains to be determined whether or not CD34^+^/KDR^+^ cells fully reflect changes in EPCs capable of arterial repair and angiogenic activity [25]. Therefore, an early hematopoietic stem cells marker, CD133, was adopted as an alternative additional marker to indicate a “true” EPCs [26, 27]. In the present time, EPCs are thought to be a heterogenous population consisting of a more primitive CD133^+^/CD34^+^/VEGFR-2^+^ subpopulation and a more mature CD133^-^/CD34^+^/VEGFR-2^+^ subpopulation. The marker CD133 (also known as AC133) is a 120-kDa transmembrane polypeptide with as yet unknown biological function. It is expressed on hematopoietic stem cells and progenitor cells from human BM, fetal liver, and peripheral blood (PB) [28]. As progenitors develop to more mature endothelium-like cells, CD133 is rapidly down-regulated [27]. The CD133^+^ cells are able to form both early and late outgrowing colonies [27]. Thus, CD133 might provide a more reliable means of defining and tracking human angioblast-like EPCs and distinguishing these from mature endothelial or monocytic cells. Recently, Friedrich *et al*. [29] have demonstrated that CD34^- ^/CD133^+^/ VEGFR-2^+^ EPCs are precursors of CD34^+^/CD133^+^/VEGFR-2^+^ EPCs with a higher potential for vascular repair. These data extend current knowledge about the heterogenous EPCs population and may have implications for the treatment of vascular disease and arterial injury. Several recently published data suggested that other populations of BM-derived, circulating, or tissue resident cells might also possess properties of EPCs. In particular, these sub-populations were characterized by expression of monocyte marker CD14, together with CD34 or VEGFR-2 [30, 31]. Furthermore, “early” isolated EPCs also displayed expression of monocyte marker (CD14, CD11b, CD11c), whereas the “late” endothelial outgrowth was CD14-negative and strongly expressed markers of mature endothelial cells [32, 33]. Hence, the definition of EPCs become more universal and indeed encompass rather heterogenous cell sub-populations of multiple origins and localization, which finally differentiate into functionally active mature endothelial cells. Despite the multiple EPCs origin, common characteristics of EPCs remain the expression of stem/ progenitor marker, their clonogenic potential (i.e. the formation of colony-forming units) and proliferative capacity (i.e. the development of late high-proliferative endothelial out-growth).

Considering the growing importance of the EPCs colonies in cardiovascular research, it is crucial to investigate the characteristics of the EPCs colony in detail. There are at least 2 morphological and functionally distinct endothelial cell populations in circulating MNCs [2]. The early spindle-like outgrowth cells possess a relatively low proliferative capacity and low ability to express mature endothelial proteins [18]. These cells presumably represent cells of different lineage, which include a subset of CD14^+^/CD34^-^ monocytic cells, which have the potential to differentiate (transdifferentiate) into endothelial-like cells under certain environmental condition in the presence of special growth factors (e.g., VEGF, fibroblast growth factor, and so on) [34]. Late “out-growth cells” show a high proliferative potential and originate predominantly from BM donors and are considered as circulating angioblasts [2]. It is important to appreciate that although monocyte-derived EPCs have a lower *in vitro* proliferation potential than hematopoietic stem cells or cord blood-derived EPCs [35], the different progenitor types seem to have a similar ability to enhance neovascularization in experimental models [18, 36,  37]. One may speculate that proliferation capacity is not the decisive factor and that the reduced proliferation of the monocyte-derived EPCs is likely to be attributable to increased release of growth factors, which may act in a paracrine manner to support angiogenesis and arteriogenesis [38]. Hur *et al*. [39] have found that a specific subset of T cells (CD3^+^CD31^+^CXCR4^+^) make up the central cluster of EPC colonies. They also found that this subset of T cells enhances EPCs differentiation and angiogenesis, resulting in neovascularization *in vivo*.

In summary, a universal single or complex EPCs marker still remains to be identified, showing the heterogeneous of endothelial precursors. As a result, both different surface markers and culture properties have been used to define EPCs.

## KINETICS OF EPCs FOR POSTNATAL NEOVASCULARIZATION

### Mobilization

Recruitment of EPCs from the BM quiescent niche has been found to be associated with the activation of proteinases such as elastase, cathepsin G, and matrix metalloproteinases (MMPs) [40]. These enzymes proteolytically cleave the extracellular matrix- or cell membrane-bound molecules responsible for EPCs’ adhesive bonds on BM stromal cells (Fig. (**1**)). These cells express membrane-bound Kit ligand (mKitL), which binds to the EPCs membrane receptor c-kit when the ligand is in its soluble form (sKitL). MMP-9 proteolytically cleaves mKitL to sKitL, which then interacts with the EPCs c-kit receptor to conduct the signal essential for BM-EPCs differentiation and migration to the peripheral blood (PB) [41] (Fig. (**1**)). One of the models used in studying the recruitment of BM hematopoietic EPCs use BM cell suppression by cytotoxic agents. This suppression does not affect hematopoietic stem cells in the G_o_ phase of the cell cycle. Therefore, these cells may serve as a cell population to reconstitute hematopoiesis and EPCs release. The introduction of cytotoxic suppression in the BM of MMP-9^+/+^ and MMP-9^-/-^ mice resulted in poor recruitment and differentiation of hematopoietic cells only in the latter group of animals [40]. Treatment of MMP-9^+/+^ mice with VEGF, stromal-derived factor-1 (SDF-1), and granulocyte colony-stimulating factor (G-CSF) caused a marked increase in the concentration of plasma sKitL compared with untreated animals [40]. The results of this experiment proved that VEGF, SDF-1, and G-CSF play significant roles in the induction of MMP-9 precursor biosynthesis, secretion, and further mobilization of BM-EPCs to the PB [41-44] (Fig. (**1**)).

### Growth Factors that Effect EPCs Function

SDF-1 and VEGF have been considered crucial in the differentiation and migration of EPCs [41-44]. Increased plasma levels of SDF-1 and VEGF are eventually accompanied by the mobilization of hematopoietic cells, among them EPCs, into the circulation (Fig. (**1**)). SDF-1 is a chemokine that binds specificially to the receptor designated as CXCR4 [43]. This receptor is expressed on the surface of hematopoietic stem cells, including EPCs [43, 45]. The SDF-1 acts as a key chemokine for the mobilization of EPCs and other progenitor cells from the BM compartment, e.g. by MMP-9-mediated cleavage of mKitL [46-48] (Fig. (**1**)). SDF-1 further mediates the recruitment of progenitor cells along hypoxic gradients and towards surface-adherent platelets after artrial injury [46-48]. Hristov *et al*. [49] revealed that blocking CXCR4 significantly reduced the adhesion of EPCs after arterial wire-injury *in vivo.* Additionally, SDF-1 mediated migration of isolated EPCs, enhanced their matrix arrest when acting as a soluble chemokine, and was further secreted by activated platelets and SMCs after arterial wire-injury [49, 50]. Within the clinical context, a dysregulation of the CXCR4 signaling in EPCs from patients with stable chronic coronary artery disease has been described [51]. Thus, the role of CXCR4 in EPCs biology appears to be more universal. Recent data has provided evidence for VEGF autocrine action in hematopoietic cells, including apoptosis protection and survival effect [42]. Granulocyte macrophage colony-stimulating factor (GM-CSF) is also proposed as a possible candidate for EPCs function regulation [52]. In a study performed by Cho *et al*., recombinant GM-CSF was able tomobilize EPCs and accelerated the re-endotheliazation process in hypercholesterolemic rabbits [52].

## MECHANISMS OF CELLULAR SENESCENCE

### Phenotype and Pathways

Cellular senescence is originally described as the finite replicative lifespan of human somatic cells in culture. Senescent cells remain viable, but they do not respond to mitogenic stimuli and their morphological characteristics and function change dramatically [53-58]. They lose their original shape, their volume increases, and they acquire a flattened cytoplasma (“fried egg” appearance)**** [59, 60]. These changes are accompanied by alterations in nuclear structure, gene expression, protein processing, and metabolism [58, 59]. These phenotypic changes of senescent cells are not observed in quiescent cells and have been implicated in aging and age-associated disease [61]. More recent data have shown that cells can enter senescence rapidly, independently of the number of cell divisions, in response to various physiological stresses (radiation, oxidative stress, lack of nutrients, DNA damage, and so on) [62-65]. This type of senescence has been termed stress-induced premature senescence [66].

Although initially believed to be a cell-culture phenomenon, cellular senescence recently was observed *in vivo* as well [67, 68]. The most common means of detecting cellular senescence is by colorimetric detection of β-galactosidase in cells under mildly acidic (pH 6.0) conditions, in contrast to the more strongly acidic conditions (pH 4.0) normally required to detect endogenous lysosomal β-galactosidase activity [69]. Other biomarkers include increased expression of p53, p21, and p16 [70-73].

Senescence is a fundamental cellular program that parallels that of programmed cellular death (apoptosis). Both molecular mechanisms restrict cellular proliferation. The reason a cell is driven to apoptosis versus senescence is not yet known [74-78]. The degree of stress [75] and cell-cycle phase [74] seem to be determining factors (eg, higher doses of oxidative stress induce apoptosis, whereas lower and long-acting doses induce senescence). Moreover, apoptosis appears to occur more easily in senescent endothelial cells, yet seems to be blocked in other senescent cell types [78]. At the same time, factors involved in senescence signaling, such as p53, are also involved in apoptosis regulation through interaction with the BCL2 family of proteins [79]. In any case, cellular senescence as a biological mechanism, as well as the role that senescence has in the living organism, is, in contrast to apoptosis, not well understood.

### Telomeres and Telomerase

Significant progress has been made in our understanding of the mechanisms underlying cellular senescence. One widely discussed hypothesis of senescence is the telomere hypotheis [80]. Telomeres are non-nucleosomal DNA/protein complexes located at the ends of chromosomes that serve as protective caps and act as the substance for specialized replication mechanisms [81-83]. As a consequence of semiconservative DNA replication, the extreme terminals in successive shortening DNA replication, the extreame terminals of the chromosomes are not duplicated completely, resulting in successive shortening of the telomeres with each cell division. Critical telomere shortening is thought to trigger the onset of cellular senescence. Thus, telomere shortening has been proposed to act as a mitotic clock that ptevents unlimited proliferation of human somatic cells. However, telomeres are also involved in stress-induced premature senescence. It seems that this second pathway initiates not because of shortening, but because of changes in telomere structure (ie, alterations in the T loop and single-stranded overhang) [84, 85]. Thus both telomere length and structural integrity are necessary for proper chromosome function and avoidance of DNA damage response and its consequent triggering of senescence.

Telomerase is a cellular reverse transcriptase which catalyzes the synthesis and extension of telomeric DNA. Telomerase activity is consistently expressed in germline cells and in the majority of malignant tissue cells and is repressed in most human normal somatic cells. Strikingly, however, telomerase activation is expressed in a highly regulated manner in certain somatic cell populations, such as lymphocytes and hematopoietic stem cells. Studies on telomerase regulation in normal somatic cells have focused on expression of the two essential components of telomerase, human telomerase RNA template (hTER) and human telomerase reverse transcriptase (hTERT). There is a good correlation between the expression of hTERT mRNA and the presence of telomerase activity in extracts from tissue culture and normal and cancer tissues, whereas hTER is expressed constitutively in both cancer and normal cells, irrespective of the status of telomerase expression. The hTERT enzyme not only produces telomeric repeats that elongates telomeres, but also prevents alterations in telomere structure, protecting the telomere cap [86-88]. Early studies reported that telomerase activity was detected in cancer cells and stem cells but not in normal somatic cells [89, 90]. However, increasing evidence has suggested that telomerase activity regulates cell proliferation in normal somatic cells by telomere lengthening or telomere length-independent mechanisms [91, 92]. Human endothelial cells and VSMCs express telomerase activity, but the activity declines with *in vitro* aging due to a decrease of hTERT, leading to telomere shortening and cellular senescence [93, 94]. In fact, induction of telomerase extends the lifespan of both endothelial cells and VSMCs [95, 96], suggesting a critical role of telomere and telomerase in vascular senescence.

## FUNCTIONAL AND ANTIOXIDATIVE CAPACITY OF EPCS

To contribute to tissue repair, EPCs and stem cells in general, have to be equipped with antioxidative defence system to survive, in necrotic and ischemic tissues. Interestingly, a high resistance to oxidative stress has been considered a characteristics feature of stem cells [97, 98]. Protection against oxidative stress by ROS is accomplished by a complex defense system composed of several antioxidative enzymes that reduce the damaging effects of ROS [99]. The most vulnerable organelles to oxidative stress are the mitochondria, because of the permanent potential for the production of superoxide anions. Superoxide anions are converted to hydrogen provided by superoxide dismutases, whereas hydrogen peroxide is detoxified by the enzymes by catalase and glutathione peroxidase. Because of the localization of MnSOD and GPx-1 in the matrix of the mitochondria, in close proximity to the production of ROS by the electron transport chain, these two enzymes are believed to be the primary antioxidant defense systems in the mitochondria. What is the specific mechanism by which ROS affect mobilzation and progenitor cell differentiation toward the endothelial lineage? Recently, two *in vivo* studies demonstrated that EPCs express high levels of MnSOD and GPx-1 [15, 16]. Galasso *et al*. [100] have addressed the role of GPx-1 for the functional capacity of EPCs *in vivo*. They investigated ischemia-induced neovascularization in GPx-1deficient mice and assessed the number and functional activity of EPCs. GPx-1-deficient mice showed a reduced blood flow recovery after hindlimb ischemia compared with their wild type. This was accompanied with reduced EPCs levels in response to the functional capacity of EPCs to migarate and promote angiogenesis *in vivo*. These findings support the knowledge that EPCs require antioxidative enzymes, especially GPx-1, for their functional capacity. However, is the reduction of EPCs in GPx-1-deficient mice caused by increased cell death or decreased differentiation or mobilization? Galasso *et al*. [100] demonstrated that EPCs derived from GPx-1-deficient mice show increased apoptosis sensitivity and decreased expression of Flk-1 are involved in the reduction of EPC levels from GPx-1-deficient mice. However, a reduced Flk-1 expression was demonstrated before and after ischemia, supporting the hypothesis that interfering with the antioxidant defense system of cells may also influence differentiation. Although the precise molecular mechanisms are unknown so far, one may speculate that the reduced bioavailability of NO reported for the GPx-1-deficient mice may contribute to the phenotype of the mice [101]. It is also important to note that eNOS-deficient mice show a similar impaired capacity to mobilize EPCs combined with a systemic dysfunction of isolated EPCs [102, 103]. The finding that GPx-1 expression is essential for EPC functions may also have clinical implications, given that patients with chronic heart failure [104] and with type 2 diabetes [105] showed a downregulation of GPx-1. This in turn may contribute to the reduced EPC numbers and function in patients with CAD and severe heart failure [106]. However, other antioxidative enzymes such as superoxide dismutase and catalases are also downregulated in these patients [105, 106]. In addition, some controversy does exist about the effects of oxidant stress on EPCs. Ingrum *et al*. [17] reported that clonogenic cord and adult blood-derived EPCs are sensitive to oxidant stress. Furthermore, EPCs treated with oxidants undergo increased apoptosis and decreased tube formation *via* apoptosis signal-regulating kinase 1 (ASK1) activation. Thus, it seems mandatory to emphasize the importance of designing therapeutic strategies to protect EPCs against oxidant stress to enhance new vessel formation.

## EFFECTS OF ATHEROGENIC FACTORS ON EPCS SENESCENCE

Clinical studies clearly demonstrate that high EPCs levels are associated with reduced cardiovascular event rates underlying the vasculoprotective action of EPCs [107, 108]. The rejuvenation of the endothelium by circulating EPCs may represent a novel approach in the prevention of atherosclerotic disease. However, limitations in therapy may come from the negative influence of cardiovascular risk factors, which are apparently overwhelming the organism’s repair mechanisms, bringing the equilibrium between vascular repair and injury out of balance (Fig. (**2**)). Cardiovascular risk factors negatively influence EPCs number and function, whereas vasculoprotection is at least in part mediated by functional active EPCs. Therefore, EPCs may present a cellular risk marker, integrating the positive and negative mediators affecting the endothelial monolayer. Multiple factors seem to be involved in the aging-associated deterioration of EPCs quantity and function. The chronic exposure to cardiovascular risk factors continuously damages endothelial cells and requires their intensive replacement. Conversely, cardiovascular risk factors possibly affect EPCs mobilization, integration in injured vascular sites, and angiogenic capacity (Fig. (**2**)). Recent studies have underlined the detrimental effects of type 1 and 2 diabetes on EPCs function [5, 109]. Loomans *et al*. [109] have demonstrated that the media from EPCs culture of type 1 diabetic patients not only possess evidence of reduced angiogenic capacity, but also contain an inhibitor for *in vitro* tube formation. Tepper *et al*. [5] reported that the proliferation and tube formation of EPCs were impaired in patients with type 2 diabetes compared with normal subjects. In both studies, decreased number and dysfunction of EPCs was inversely related to the levels of hemoglobin A1c, implying that the degree of glycemic dysregulation was associated with EPCs pathophysiology. Although these studies have clarified an adverse effect of DM on the functional activity of EPCs, the underlying mechanisms remain unsolved. The EPCs dysfunction may also be result of their accelerated senescence. We showed that hyperglycemia (HG) increases the rate of EPCs senescence, which effect is inhibited by an inhibitor p38 MAPK, SB203580 [110]. We also have demonstrated that high glucose (HG) levels can accelerate the p38 MAPK pathway in EPCs [110]. Seeger *et al* [111] have demonstrated that the redunction of EPCs induced by high glucose (HG) *in vitro* is associated with a profound upregulation of p38 mitogen activated protein kinase (MAPK) phosphorylation and is completely blocked by p38 inhibitors. Furthermore, EPCs cultivated from patients with CAD show an increased p38 phosphorylation compared with EPCs from healthy control subjects. Interestingly, they have shown that HG further augment the phosphorylation of the p38 downstream kinase stress-activated kinase (MSK)1 and the transcription factor camp-responsive element-binding protein (CREB). Several studies demonstrate that p38 MAPK blockade inhibition is associated with increased angiogenesis [112-114]. Because NAD(P)H oxidase activation promotes p38 MAPK phosphorylation [111], it is tempting to speculate that increased p38 MAPK phosphorylation may represent an additional potential pathway whereby NAD(P)H oxidase may alter EPCs function in DM. Therefore, p38 MAPK inhibitors and/or NAD(P)H inhbition might be a promising tool to augment the yield of ex-vivo-expanded EPCs for cell therapy, specially for the patients with DM. In this context, Sorrentino *et al*. [115] have demonstrated that short-term *in vivo* rosiglitazone treatment in diabetic subjects reduced EPCs NAD(P)H oxidase activity and restored NO availability, suggesting that PPAR-γ agonist exerts a direct on NAD(P)H oxidase in diabetic EPCs. Of note, *in vitro* treatment with the PPAR-γ agonist pioglitazone prevented oxidative stress-induced apoptosis in human EPCs, further suggesting a role of PPAR-γ for EPCs function [116]. We have also shown that pioglitazone reduces Ang II-induced acceleration of senescence in EPCs [117]. Collectively, therapeutic interventions that improve vascular activity in EPCs by PPAR-γ agonists may have tremendous potential for the treatment of cardiovascular diseases.

The number of circulating EPCs in patients with CAD has been shown to decline with increasing age [3]. Furthermore, following coronary artery bypass grafting, EPCs mobilization is significantly impaired in older individuals compared with younger patients [118]. Besides changes in EPCs levels, the function of EPCs from older individuals also appear to be disrupted, based on *in vitro* examination of EPCs survival, proliferation, and migration [3, 119]. These results strongly suggest that age is an important determinant of EPCs function and further support the hypothesis that changes in EPCs function with age contribute to the impairment of cardiovascular repair mechanisms in the aging host. The age-associated impairment of cardiac angiogenic capacity in older mice, estimated as neovascularization of cardiac allografts, can be restored by implantation of BM-derived EPCs from young adult animals [120]. Progression of atheroscleorsis in apolipoprotein E^-/-^ mice with persistent hypercholesterolemia seems delayed by chronic administration of BM-derived progenitor cells from young mice [120]. This treatment was much less effective when donors were older animals with atherosclerosis, indicating that progressive age-dependent reduction in EPCs may accelerate the development of atherosclerosis, particularly in the presence of hypercholesterolemia [120].

Reduced levels of angiogenic and mobilizing cytokines have been related to age-dependent impairment of EPCs mobilization *in vivo*. Indeed, vascular endothelial growth factor (VEGF) and NO production have been reported to decrease with age [118-120]. Specifically, eNOS expression and subsequent NO production are crucial in EPCs mobilization (Fig. (**1**)) [121, 122]. In this context, eNOS is a central downstream mediator in VEGF-signaling pathways [123-125]. Moreover, we and others have shown that ox-LDL, which accumulates with age, also suppresses eNOS expression and impairs EPCs survival and function [126, 127]. We showed that ox-LDL accelerates the onset of EPCs senescence, which leads to impairment of proliferative capacity and network formation [127]. However, the mechanisms by which ox-LDL accelerates the onset of EPCs senescence remain unclear. We also demonstrated that, in the presence of VEGF, ox-LDL reduces the number of adherent EPCs through dephosphorylation of the Akt kinase on Ser^473^ in EPCs [128]. Dimmeler *et al*. [129] showed that VEGF, as well as statins, induces EPCs differentiation *via* the PI3-K/Akt pathway, as showed by the inhibitory effect of pharmacological PI3-K blockers or the overexpression of a dominant negative Akt construct. Interestingly, Breitschopf *et al*. [130] showed that a dominant-negative Akt significantly reduced telomerase activity in HUVECs. Therefore, it is possible that ox-LDL accelerates the onset of EPCs senescence through telomerase inactivation, which may be related to inactivation.

## POTENTIAL ROLE OF OXIDATIVE STRESS IN EPCS SENESCENCE IN ACTIVATION OF RENIN-ANGIOTENSIN SYSTEM

### Ang II and Oxidative Stress

Ang II increases oxidative stress, inflammation, and alters endothelial function [131, 132]. The principle sources of ROS in the human vasculature is NAD(P)H oxidase, activated by a number of proatherogenic stimuli including Ang II. Overall NAD(P)H oxidase activity in cells is achieved by several components. These included cell-membrane-associated p22phox and gp91phox (or gp91phox [nox2] homologues, nox1 and nox4), as well as cytosolic p47phox and p67phox [132]. Vascular NAD(P)H oxidase is upregulated by vasoactive factors, stretch, shear stress and pulsatile strain [132]. At low concentrations ROS serve a physiological role as signalling molecules involved in endothelial function and vascular contractility [131]. However, pathological increases in ROS results in a plethora of effects that damage the vessel wall [133].

### Ang II and EPCs Senescence

We have shown that in animal models of hypertension, as well as in subjects with essential hypertension, EPCs become precociously senescent and dysfunctional [134]. Higher blood pressure is associated with lower EPCs levels in the general population [135], and in diabetic subjects [136]. Hyperreactivity of the renin-angiotensin system (RAS) has been recognized as one link between hypertension and altered EPCs biology. Bahlmann *et al*. [136] documents that angiotensin receptor antagonists increase the number of EPCs in patients with type II diabetes mellitus. This effect seems to be a class effect, because they have demonstrated it with standard doses of 2 long acting ARBs (olmesartan or irbesartan). In contrast, in patients treated with standard antihypertensives, they did not observe any effects of EPCs. Ramipril is an angiotensin-converting enzyme (ACE) inhibitor used to reduce RAAS activation in patients with stable CAD. Min *et al*. [137] showed that increased numbers of EPCs could be cultured from ramipril-treated patients with stable CAD and that ACE inhibition resulted in improved functional properties like adhesion, proliferation, migration, and *in vitro* vasculogenesis assay, independent of any impact on blood pressure. These results show that EPCs are sensitive to Ang II signalling and that this should indeed impact on number and function. Our group has shown that Ang II increases the rate of senescence of EPCs and that this appears to be a consequence of its ability to stimulate expression of gp91phox and thus O_2_^-^ formation [138]. In addition, we have demonstrated that the ability of Ang II to induce senescence also involves the suppression of telomerase [138]. Our group also noticed an expected increase in formation of a marker of oxidative stress-peroxynitrite, which is formed of O_2_^-^ with NO in the Ang II-treated EPCs [138]. Increased O_2_^-^ production is a feature of Ang II-dependent hypertension [139]. Thus, under conditions of Ang II excess, Ang II most likely contributes to the decline in formation of the vascular endothelium at least in part by the mechanisms *via* Ang II-induced EPCs senescence (Fig. (**3**)). On the other hand, it has been demonstrated that Ang II and angiotensin peptides promote hematopoietic progenitor cell proliferation and hematopoietic recovery after radiation therapy and chemotherapy [140]. Murohara *et al* reported that the Ang II-AT1 receptor pathway plays an important role in angiogenesis associated with ischemia and tumor growth [141, 142]. These results appear to be contradictory to protective effects of RAS suppression on EPCs function. Recent studies suggested that the intracellular redox state is a critical modulator of the balance between self-renewal and differentiation in dividing precursor cells and that anti-oxidant may preserve their stemness [143]. It is plausible that a reduction in oxidative stress resulted in restoration of the impaired faction of EPCs in spontaneous hypertensive rats as well as patients with metabolic disorders, although it remains to be determined whether RAS inhibition stimulates EPCs function under physiological conditions in healthy subjects.

## PHARMACOLIGIC MODULATIONS ON EPCS SENESCENCE

### HMG-CoA Reductase Inhibitors (Statins)

Pharmacologically, statins have been shown to enhance EPCs-mediated angiogenesis in models of ischemic tissue injury. *In vivo*, statin treatment increases the numbers of circulating EPCs and enhances both neovascularization in corneal assays and reendotheliazation of injured vessels, promoting incorporation of labeled BM-derived cells into these vessels [144-146]. Mechanistically, statin treatment *in vitro* appears to inhibit EPCs senescence, *via* induction of telomere repeat binding factor-2, which inhibits induction of the DNA damage checkpoint-kinase 2 [147]. Simvastatin activates the serine-threonine kinase Akt in endothelial cells, promoting endothelial cell survival and migration [148]. Akt also acts downstream of VEGF and may therefore represent a key regulator of VEGF-mediated neovascularization [149]. Thus, these data suggest that statin therapy may constitute an important approach in the development of strategies to improve EPCs survival and function and to improve cardiac repair pathways in the aging population. Indeed, the TOPCARE-AMI clinical trial demonstrated that the treatment of ex vivo cultured blood-derived progenitor cells with atorvastatin was found to be safe and potentially effective for the enhancement of cardiac regeneration [150].

### Ang II Receptor Blockers (ARBs)

ARBs have been shown to be antioxidant and vasoprotective through downregulation of vascular NAD(P)H oxidase expression in patients with CAD [151]. Treatment with either an ACE inhibitor or ARB lowered levels of vascular superoxide. Bahlmann *et al*. [136] reported that ARBs increase the number of EPCs in patients with type II diabetes mellitus. Our group reported that Ang II accelerates EPCs senescence *via* the AT_1_ receptor and through induction of oxidative stress [138], (Fig. (**3**)). Exposure of cultured EPCs to Ang II significantly accelerated the rate of senescence compared with a control and impaired proliferative activity. We also showed that Ang II-induced EPCs senescence was significantly inhibited by pretreatment with either valsartan or superoxide dismutase (SOD) [138]. Ang II also significantly diminished telomerase activity, and this effect was significantly reduced by pretreatment with either an AT1 receptor antagonist, valsartan, or SOD [138]. In addition, Yao *et al*. [152] reported that EPCs colony formation was markedly lower in SHR-SP than in WKY rats. Losartan improved the reduced colony formation with inhibition of oxidation from SHR-SP by reducing expression of gp91phox, p22phox, and p47phox. Trichlormethiazide did not affect the reduced colony formation in EPCs. Thus, EPCs function was altered in Ang II-dependent hypertension with oxidative stress. These data indicate that EPC has an Ang II-generating system that accelerates senescence of EPCs and may directly contribute to vascular injury in hypertension.

### Estrogens

No direct studies of effect of estrogen therapy on EPCs in humans are available, but increased blood estrogen levels in women do correlate with numbers of circulating EPCs [130]. In an animal carotid injury model, estradiol treatment showed stimulatory effects on EPCs mobilization, proliferation, mitogenic and migratory activity, as well as inhibited EPCs apoptosis [123]. Mechanistically, we have demonstrated that estrogen augments differentiation and delays the onset of the senescence in BM-EPCs from SHR/Izm, accompanied with telomerase activation *via* up-regulation of hTERT mRNA in a PI3-K/Akt dependent manner [153]. Importantly, the inhibition of BM-EPCs senescence by estrogen *in vitro* resulted in improved functional activity of BM-EPCs [153]. We showed that 17β-estradiol dose-dependently inhibited the senescence of cultured human PB-MNCs by increasing the catalytic activity of telomerase [154]. We also showed that telomerase activity in EPCs is upregulated by treatment with 17β-estradiol. Furthermore, we have demonstrated that this activation accompanied upregulation of the hTERT mRNA [154]. We speculated that estrogen delays the onset of senescence through telomerase activation, which may be related to estrogen-induced upregulation of the expression of hTERT. However, with regard to senescence, the structure of the telomere appears at least as important as its absolute length, in relation to telomere function [155]. In addition, we can not rule out the possibility that a telomere-independent mechanism regulates replicative senescence. Further studies are required to elucidate the mechanisms underlying the inhibitory effects of 17β-estradiol on senescence in EPCs. Importantly, the inhibitory effects of 17β-estradiol on EPCs senescence may not be of only a beneficial nature. Telomere shortening is probably one of the body’s anticancer mechanisms, and estrogen is considered to be a potentially cancer-promoting substance. Indeed, estrogens increase the incidence of various malignancies [156]. It is tempting to speculate that the induction of neoplasia during estrogen treatment might be dependent on the effects of EPCs.

## CONCLUSIONS AND IMPLICATIONS

Recent data show that the vascular regenerative potential of patients with cardiovascular risk factors may be impaired as a consequence of reduced number and function of circulating EPCs that can support endothelial maintenance and ischemia-induced neovascularization. Recent studies have demonstrated a critical role of oxidative stress in the EPCs dysfunction. Notably, oxidative stress-induced EPCs premature senescence is involved in this process. Autologous transplatation of progenitor cells that are affected by cardiovascular risk factors, may not only be hampered by a dysfunctional nature of these cells but in fact may stimulate proatherogenic mechanisms, such as monocyte recruitment or vascular smooth muscle cell proliferation. Therefore, attention should be paid to transplantation of autologous BM cells or circulating EPCs not to promote atherosclerosis. The therapeutic goal must be the rebalancing between endothelial injury and repair. In the future, the use of a vascular repair index may be important for choosing therapy strategies with a maximized benefit for the patient. With this knowledge in mind, we need to search for more effective proregenerative therapeutic strategies not only for neoangiogenesis but, more importantly, for regeneration of the dysfunctional vascular wall, which represents the common trunk for all cardiovascular diseases.

## Figures and Tables

**Fig. (1). Mobilization, recruitment, and differentiation of human, bone marrow-derived angiogenic progenitor cells.**
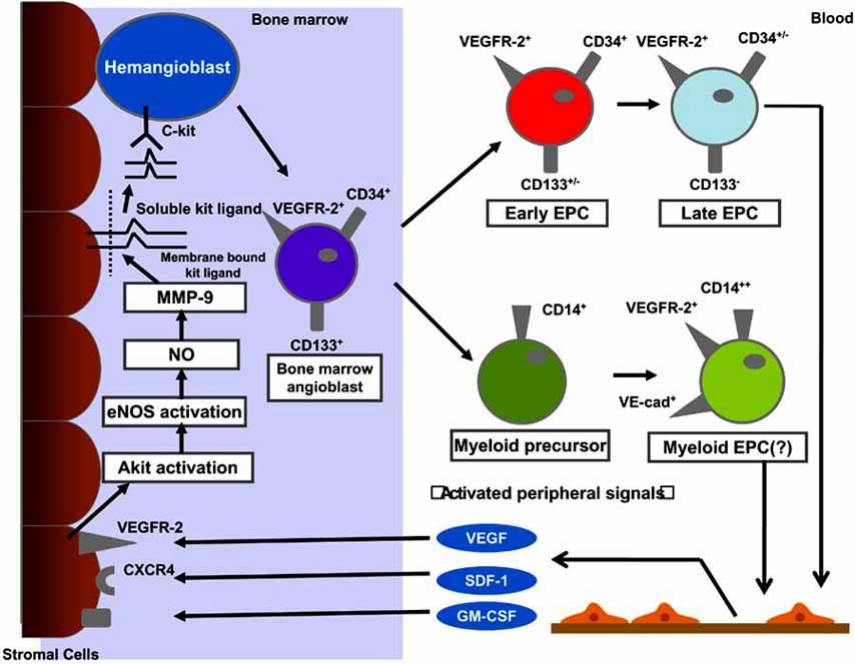
Hemangioblast, originated from hematopoietic cell, is resident in bone marrow niches, in a quiescent state. The stimulation by circulating cytokines induces the activation of matrix metalloproteinase-9 (MMP-9) through an Akt, nitric oxide dependent pathway. MMP-9 promotes the transformation of membrane bound Kit-ligand to a soluble Kit-ligand. This activation is followed by detachment of early c-Kit+ progenitor cells from the bone marrow stromal niche and their subsequent movement to the vascular zone of the bone marrow. An important regulation is VEGF and SDF-1, which binds to its receptor VEGFR-2 and CXCR4, respectively, thus mediating further maturation of the cascade *hemangioblast-angioblast-early endothelial progenitor cells (EPC)-late EPCs*. Bone marrow-derived EPCs are of hematopoietic origin and possibly derive from the hemangioblast. These early progenitors (CD133^+^/CD34^+^/VEGFR-2^+^/CD14^-^) represent a small population with proliferative potential, capable to give rise to late endothelial outgrowth. Cells of myeloid origin (CD14+) may also trans-differentiate into endothelial cells and secret angiogenic factors, but their proliferative potential is limited and they did not generate a stable late outgrowth.

**Fig. (2). Oxidative stresses on endothelial progenitor cells (EPCs) in cardiovascular diseases.**
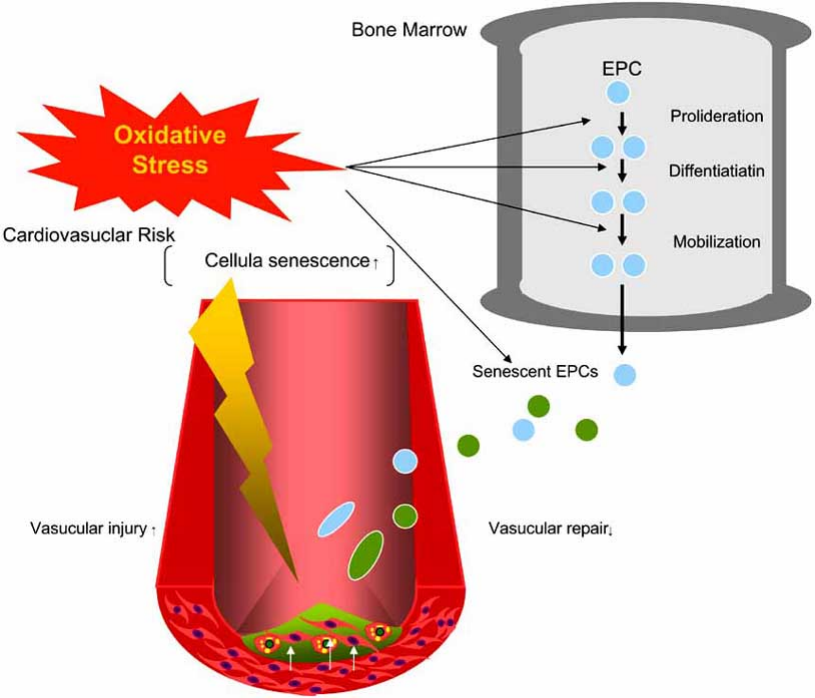
EPCs repair cardiovascular damage. Oxidative stress caused by dyslipidemia, diabetes mellitus, or hypertension interferes with the ability of EPCs proliferation, differentiation, and mobilization in bone marrow. Oxidative stress also induces EPCs senescence. These negative effects of oxidative stress on EPCs number and function bring the equilibrium between cellular repair and injury out of balance, resulting in the progression of cardiovascular damages.

**Fig. (3). Potential mechanisms of Ang II-induced EPCs senescence.**
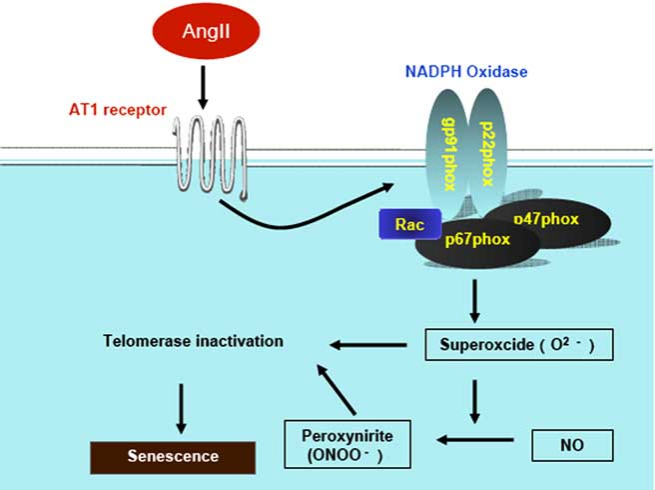
Ang II stimulates gp91^phox^ expression, a subunit of NADPH oxidase, via the angiotensin type 1 (AT_1_) receptor, which leads to the increase in superoxide (O_2_^-^). Furthermore, peroxynitrite is formed form the inteaction of O_2_^-^ with nitric oxide (NO). Both superoxide and peroxynitrite inactivate telomerase activity, which induces the impairment of telomere structure integrity, resulting in senescence.
